# Exploring smallholder farmers’ access and participation in the Home Grown School Feeding Programme in selected counties of Kenya

**DOI:** 10.3389/fpubh.2024.1476888

**Published:** 2025-01-22

**Authors:** Joyce Kamau, Collins Okoyo, Tabitha Kanyui, Charles Mwandawiro, Samrat Singh, Lesley Drake

**Affiliations:** ^1^Partnership for Child Development (PCD), Imperial College London, Nairobi Office, Nairobi, Kenya; ^2^Eastern and Southern Africa Centre of International Parasite Control (ESACIPAC), Kenya Medical Research Institute (KEMRI), Nairobi, Kenya; ^3^Department of Epidemiology, Statistics and Informatics (DESI), Kenya Medical Research Institute (KEMRI), Nairobi, Kenya; ^4^Partnership for Child Development (PCD), Imperial College London, London, United Kingdom

**Keywords:** Home Grown School Feeding Programme, smallholder farmers, farmer-based organizations, markets access, mobile phone platform

## Abstract

**Introduction:**

Smallholder farmers (SHFs) produce 80% of the total agricultural output in Kenya. The Home Grown School Feeding Programme (HGSFP) was designed to address short-term hunger among primary school children from food-insecure households, enhancing access to primary education while providing a market to SHFs through local procurement of food for schools. This study investigated SHF access and participation in the HGSFP market and the uptake of a mobile phone platform (MPP) in HGSFP procurement in Tharaka Nithi, Kitui and Kilifi Counties of Kenya.

**Methodology:**

Descriptive cross-sectional study design was utilized and data were collected from SHFs, school teachers and farmer-based organizations (FBOs) within the schools’ locality using semi-structured questionnaires. A total of 378 SHFs, 92 FBOs and 70 school teachers were interviewed for the study. Data were analyzed using Stata version 16.

**Results:**

The study revealed that SHFs (22.8%) and FBOs (37.5%) gained access to HGSFP market and sold produce of maize (92.9%) and beans (91.4%). The main channel used by SHFs to sell produce to schools was through the FBOs (61.6%) amidst challenges of lack of surplus to sell (53.2%), low prices (50.9%) and poor transport infrastructure (23.6%). HGSFP schools purchased most of their food requirements from traders/brokers through manual tendering (65%). The uptake of the MPP for procurement of food by HGSFP schools and FBOs was embraced and promising and was rated as faster to use (76.8%) and more transparent in HGSFP procurement (44.6%).

**Conclusion:**

The study concluded that local procurement opportunities through FBOs were underutilized. We recommend more capacity-building support for SHFs and FBOs to increase their production and give them better opportunities to be key participants in the HGSFP market and other structured markets. The MPP should be adopted for the procurement of food for school meals for transparency and accountability. To maximize its benefits, it should be inclusive of all market players, especially traders/brokers and sufficient training should be provided to all stakeholders to participate fully in the HGSFP market.

## Introduction

Kenya is largely an agricultural country with the sector contributing approximately 33% to the country’s Gross Domestic Product (GDP). The agriculture sector employs 70% of the rural population with the main players being SHFs, who make up about 7.5 million (1). Food production activities by SHFs in Kenya are conducted on land averaging 1–5 acres (less than 2 ha). This is on the background of Kenya’s 28 million hectares of agricultural land which makes up 48% of the total land area in the country (2). However, SHFs use only 60% of their land for agricultural production (2). Notably, 84% of the land in Kenya is arid and semi-arid and unsuitable for rain-fed crop production with limited production of some crops. Only 16% of the land receives adequate and reliable rainfall and has high and medium agricultural potential. Small-scale farming produces 75% of total agricultural output and 70% of marketed produce (3).

However, the productivity of SHFs is affected negatively by challenges such as poor access to productive resources (storage facilities, access to finance, low use of improved modern technology), poor market linkages (low bargaining power, selling produce at farm gate prices), poor rural infrastructure in terms of road networks and transportation; and climate change (4). Therefore, there is a need to support SHFs to enhance their production. One way of supporting them is through collective action where SHFs are organized into FBOs through which they can access production inputs, training on good agricultural practices including climate-smart farming methods, and improved use of modern agricultural equipment (5, 6). Secondly, capacity building for SHFs may be achieved through public procurement particularly HGSFP which provides a unique platform to link the demand for school meals to SHF production (7, 8). This may lead to a diversified food basket, localized supply chains, and sustained demand throughout the year. A study by Prifti and Grinspun among SHFs in Zambia noted that SHFs have greater success when increased demand from HGSF programme is accompanied by interventions to increase production thus supply (9). This support is also necessary to increase production of nutritious crops for the programme and training in procurement procedures such as how to win tenders and price negotiation as well as having smallholder-friendly procurement regulations (10).

The HGSFP was launched in 2003 when African governments included locally sourced school feeding programmes in pillar 3 of the Comprehensive African Agriculture Development Programme based on the New Partnership for Africa’s Development vision for nationally owned sustainable school feeding programmes (7). The main goal of HGSFP is to link school feeding to agricultural development by purchasing and using locally produced food in feeding school children (8, 11). In Kenya, HGSFP was launched in 2009 to address short-term hunger among primary school children from food-insecure households, enhancing access to primary education and providing a market to SHFs (12). The programme is regarded as a safety net measure for malnourished children in Kenya (13).

In the HGSFP, funds are sent directly to beneficiary schools where the school meals committees are responsible for procuring food locally. The programme is well positioned to improve the nutritional status of school children as well as champion investments in rural procurement of food from local smallholders thereby improving SHFs’ incomes and stimulating local agricultural growth (14, 15). Through this, the local food systems are strengthened, local production is encouraged and jobs are created across the school meal value chain which may lead to the consumption of locally produced food by schoolchildren (16). Additionally, the school meal programme has led to increased enrollment, attendance, retention and other educational achievements among learners in Kenya (17, 18).

However, studies show limited access to the HGSFP by SHFs and failure to promote local sourcing of food by the participating schools (19, 20). Market linkages between SHFs and schools have been weak amidst challenges experienced by farmers including tough procurement conditions for SHFs (21). The low productivity among SHFs is coupled with challenges in securing a stable supply of locally produced food due to adverse climatic conditions (10, 18). In addition, despite the variety of crops produced by SHFs, schools have not fully utilized them to provide diverse diets to school children (22). The few studies on the impact of HGSFP on SHFs have produced mixed results.

This paper explores the extent to which SHFs and FBOs accessed and participated in the HGSFP market in Tharaka Nithi, Kitui and Kilifi counties of Kenya, the range of crops and food items that were supplied to HGSFP schools and the use of MPP in the procurement process for HGSFP. The following sections of this manuscript present the methodology employed in the study, findings from the study, discussion of the findings and conclusions.

## Methodology

### The intervention

The Partnership for Child Development (PCD) partnered with the Government of Kenya through the Ministry of Education to implement the project “An Investment in Human Capital and Rural Economies” from March 2019 to June 2023. The project aimed to build the capacity of SHFs organized in FBOs to enhance the production of food commodities and to link them to the HGSFP market through the MPP. The project also aimed to enhance the transparency and accountability of the procurement system of HGSFP through the use of mobile technology. The project was implemented in three counties where HGSFP was already being implemented by the government. The project counties were Tharaka Nithi in central Kenya, Kilifi County in the coastal region and Kitui County in the eastern region of Kenya. The project covered 100 primary schools across the three counties with 50,000 pupils and 100 FBOs with 5,000 SHFs.

Through the project, market linkages between schools and FBOs were created through the MPP to address the difficulty of access to markets by SHFs. To support the SHFs and schools, 3 sets of interventions were implemented: set 1 included the development of the MPP, training teachers and FBOs on the use of MPP in the HGSFP procurement procedures; set 2 comprised of training SHFs on Good Agricultural Practices, Post-Harvest Handling, distribution of certified seeds including maize and high iron and zinc beans, farm tools and equipment, establishment of demonstration farms to encourage the adoption of certified seeds and good agricultural practices and pest control. Set 3 of the intervention was on aggregating food crops among SHFs to raise sufficient quantities of food for schools. Regular monitoring was undertaken throughout the project period with key indicators for FBOs being aggregated quantities of produce, sales to HGSFP schools, quality control measures practiced by FBOs, and the status of storage facilities. The indicators monitored for schools were the number of schools that published tenders on the MPP, the number of schools that procured food from FBOs through the MPP and the type and quantity of food procured through the MPP. At the end of the project, a study was conducted to determine the extent to which schools procured food from SHFs and FBOs using the MPP, SHF production and type and quantity of foods sold to schools.

### Study design and study site

Data were collected between April and June 2023 in the rural settings of Kitui, Kilifi, and Tharaka Nithi Counties of Kenya.

Kitui County is located in the eastern part of the country. It is about 30,496 (km^2^) in size with a population of 1,136,187 people (23). The climate is semi-arid with approximately 400–1,000 milliliters of precipitation. There are two rain seasons, long rain season occurs around March & April, and the short rain season is around October, November and December (24).

Kilifi County is one of the six counties in the coast region. It has an area of 12,609.7 km^2^ and a population of 1,453,787 (23). The average annual rainfall ranges from 300 mm in the hinterland to 1,300 mm in the coastal belt and the temperatures range between 21°C and 30°C in the coastal belt and between 30°C and 34°C in the hinterland. The main food crops are maize, bananas, cowpeas, green grams and upland rice (25).

Tharaka Nithi County borders Kitui County to the East and South East with an area of 2,609km^2^ and a population of 393,177 (23). The temperatures range between 14°C to 30°C with a high of 40°C during the dry season. Rainfall ranges from 500 mm to 2,200 mm with the long rains occurring from April to June. The main food crops are maize, cowpeas, pigeon peas, and green grams (26).

### Study population and sample size

The study targeted 5,000 SHFs, 100 FBOs and 100 primary schools across the three counties. The respondents for the study were selected using simple random sampling. Sample sizes were determined in the following manner:

### Individual interviews with smallholder farmers

The study randomly sampled the SHFs in the study counties as per the following formula by Yamane (27) and based on the total population size of SHFs enrolled in the project.


$$n_{o}=\left[\frac{N}{1+N{\left(e\right)}^{2}}\right]=\left[\frac{5000}{1+5000{\left(0.05\right)}^{2}}\right]=370\;\mathrm{SHFs}$$

Since the total number of SHFs was below 10,000, the minimum sample size was adjusted downwards as follows.


$$n=\frac{n_{o}}{1+\left[\frac{n_{o}-1}{N}\right]}=\frac{370}{1+\left[\frac{370-1}{5000}\right]}=345\;\mathrm{SHFs}$$

Where, $n_{o}$ and n are the initial minimum and adjusted minimum required sample size respectively, N is the total population size of the targeted SHFs in the three counties, and e is the margin of error assumed as 5%.

Additionally, 20% non-response rate was added to raise the final sample size to 414 SHFs. The final sample size was distributed among the counties using the probability proportional to size (PPS) strategy as per the formula, $\left[\frac{N_{h}}{N}\times n\right]$ , where $N_{h}$ is the number of smallholder farmers in a county, N the total number of smallholder farmers in the three counties, and n is the total sample size. The values of $N_{h}$ in this study were Kilifi County (1,275), Kitui County (2,635) and Tharaka Nithi County (1,102). This resulted in a sample size of 105 SHFs in Kilifi County, 218 in Kitui County and 91 in Tharaka Nithi County. Simple random sampling was used to select respondents for the study within the counties.

### Interviews with farmer based organizations

The Project delivered interventions directly to 100 FBOs spread across the three counties: Kitui County (36), Tharaka Nithi County (34) and Kilifi County (30). A census of all participating FBOs was deemed necessary and practical to obtain the relevant data. Therefore, the chairperson and secretary of each FBO were purposefully requested to participate in the study being the custodians of group records and all the relevant information required from the FBO.

### Interviews with school head teachers

A total of 100 schools spread across the three project counties participated in the project. The schools were similarly distributed as Kitui County (36), Tharaka Nithi County (34) and Kilifi County (30). However, 94 schools received cash disbursements from the government to procure food during the period under review. The researcher intended to include all the 94 schools in the study, however, only 70 schools participated. Data for each school were obtained from the head teacher supported by the teacher in charge of school meals through a questionnaire. The teachers were purposively selected to respond to interviews being the key implementers of the HGSFP in the participating schools.

### Data collection procedures and statistical analysis

Data were collected by a team of trained enumerators who were recruited from the respective counties in collaboration with project partners. The field team was composed of college degree and diploma holders and was supported and coordinated by trained supervisors.

The primary data were collected from SHFs, FBO leaders and head teachers of participating schools in the three project counties using semi-structured questionnaires. Data was collected from the SHFs on socio-demographic and economic factors, types of crops produced and marketed during the previous season (2022) and the markets accessed in that period. The FBO leaders were interviewed on the organizational structure of FBOs, training, capacity building and other services offered by FBOs, production and marketing of members’ produce, and the use of MPP in marketing. Data from the schools included the types of food purchased for school meals, the procurement process for school meals and the use of MPP in the procurement process.

The open data kit (ODK) was used to collect data via mobile phones. The ODK suite was utilized to prevent human errors, ambiguity and inefficiencies associated with the traditional method of data collection as well as to improve the overall quality of data. Data were analyzed using Stata version 16.0. Descriptive statistics including percentages and standard deviations were used to analyze the quantitative data.

### Ethics statement

Permission to conduct the survey was obtained from the Kenya Ministry of Education at the national level. Further authorization to conduct the study in the project counties was obtained from the County Directors of Education in the respective counties before any data collection procedures began. Authorities at the sub-county and ward levels were also informed about the study. All the study participants gave written informed consent to participate in the study.

## Results

### Socio-demographic and socio-economic characteristics of smallholder farmers

Out of the 414 SHFs sampled, 378 agreed to participate in this study, translating to a return rate of 91.3%. The majority of the participants were females (71.4%) and the mean age of the participants was 48.4 years (standard deviation (SD): 13.2 years, Range: 20–90 years). The highest formal level of education attained by most participants was upper primary (51.6%) followed by secondary education (23.5%) (Table 1). The mean size of households was 5.7 persons (SD: 2.5, Range: 1–15) and the main source of income was farming (75.7%) followed by business (11.4%) with an average monthly income of Ksh 10,209 (SD: 10,750, Range: 0–100,000) (Table 1).

**Table 1:** Socio-demographic and socio-economic characteristics of smallholder farmers.

Factors	Overall no. of farmers	No. of farmers by county (%)		
		Kitui	Tharaka Nithi	Kilifi
No. of farmers (%)	378 (100.0)	216 (66.9)	107 (33.1)	55 (14.6)
Sex (%)				
Male	108 (28.6)	47 (21.8)	37 (34.6)	24 (43.6)
Female	270 (71.4)	169 (78.2)	70 (65.4)	31 (56.4)
Mean age in years (SD, min-max)	48.4 (13.2, 20–90)	50.7 (13.8, 21–90)	43.6 (11.3, 20–77)	48.8 (12.1, 25–74)
Level of education (%)				
None/did not attend school	30 (7.9)	25 (11.6)	2 (1.9)	3 (5.5)
Lower primary (1–4)	25 (6.6)	20 (9.3)	0	5 (9.1)
Upper primary (5–8)	195 (51.6)	106 (49.1)	57 (53.3)	32 (58.2)
Secondary	89 (23.5)	51 (23.6)	26 (24.3)	12 (21.8)
College/University	39 (10.3)	14 (6.5)	22 (20.6)	3 (5.5)
Mean size of HH (SD, min-max)	5.7 (2.5, 0–15)	5.7 (2.6, 1–15)	5.2 (2.1, 1–12)	6.5 (2.9, 0–13)
Main source of income (%)				
Business	43 (11.4)	27 (12.5)	7 (6.5)	9 (16.4)
Casual	31 (8.2)	24 (11.1)	5 (4.7)	2 (3.6)
Farming	286 (75.7)	157 (72.7)	89 (83.2)	40 (72.7)
Salaried	12 (3.2)	3 (1.4)	6 (5.6)	3 (5.5)
Other	6 (1.6)	5 (2.3)	0	1 (1.8)
Average monthly income in KES (SD, min-max)	10,209 (10,754, 0–100,000)	10,224 (10,323, 0–60,000)	10,034 (9,606, 500–60,000)	10,491 (14,205, 10–100,000)
Disc plough	5 (1.3)	2 (0.9)	3 (2.8)	0
Ox plough	178 (47.1)	137 (63.3)	41 (38.3)	0
Tiller	5 (1.3)	1 (0.5)	4 (3.7)	0
Planter	6 (1.6)	3 (1.4)	3 (2.8)	0
Irrigation equipment	75 (19.8)	29 (13.4)	44 (41.1)	2 (3.6)
Motor cycle	88 (23.3)	41 (19.0)	35 (32.7)	12 (21.8)
Sheller	9 (2.4)	6 (2.8)	3 (2.8)	0
Drier	16 (4.2)	16 (7.4)	0	0
Family own land (%)				
Yes	368 (97.4)	213 (98.6)	103 (96.3)	52 (94.6)
No	10 (2.7)	3 (1.4)	4 (3.7)	3 (5.5)
Average size of land owned by family in acres (SD, min-max)	5 (5, 0.5–50)	5.8 (6.0, 0.5–50)	3.5 (2.5, 0.5–13)	4.5 (3.4, 0.5–12)
Family has title deed (%)				
Yes	208 (55.0)	136 (63.0)	44 (41.1)	28 (50.9)
No	170 (45.0)	80 (37.0)	63 (58.9)	27 (49.1)

In terms of ownership of assets, 97.4% of the SHFs owned land on which they lived and carried out their farming activities. The average size of land ownership was 5.5 acres. Further, slightly more than half the participants (55.0%) had title deeds indicating land ownership (Table 1). Additionally, more than half of the households (63.2%) were connected to electricity and most of the respondents owned mobile phones (95.8%). The most common agricultural assets among the SHFs were ox ploughs (47.1%) and irrigation equipment (19.8%). The motorcycle (23.3%) was a commonly owned asset among the SHFs (Table 1).

### Crops produced and sold last season by smallholder farmers

**Figure 1:**
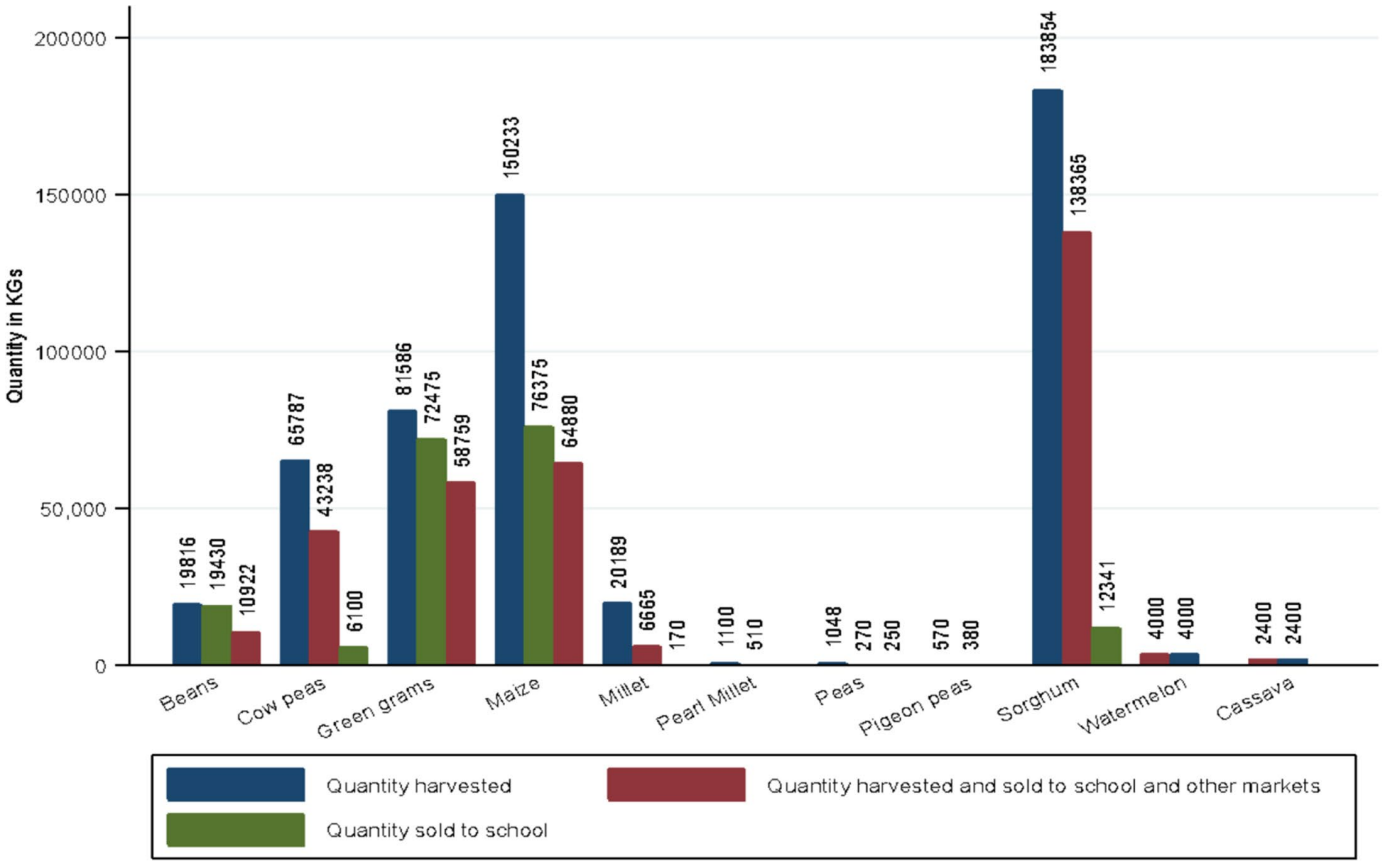
Quantity of crops produced and sold to schools and other markets last season.

**Figure 2:**
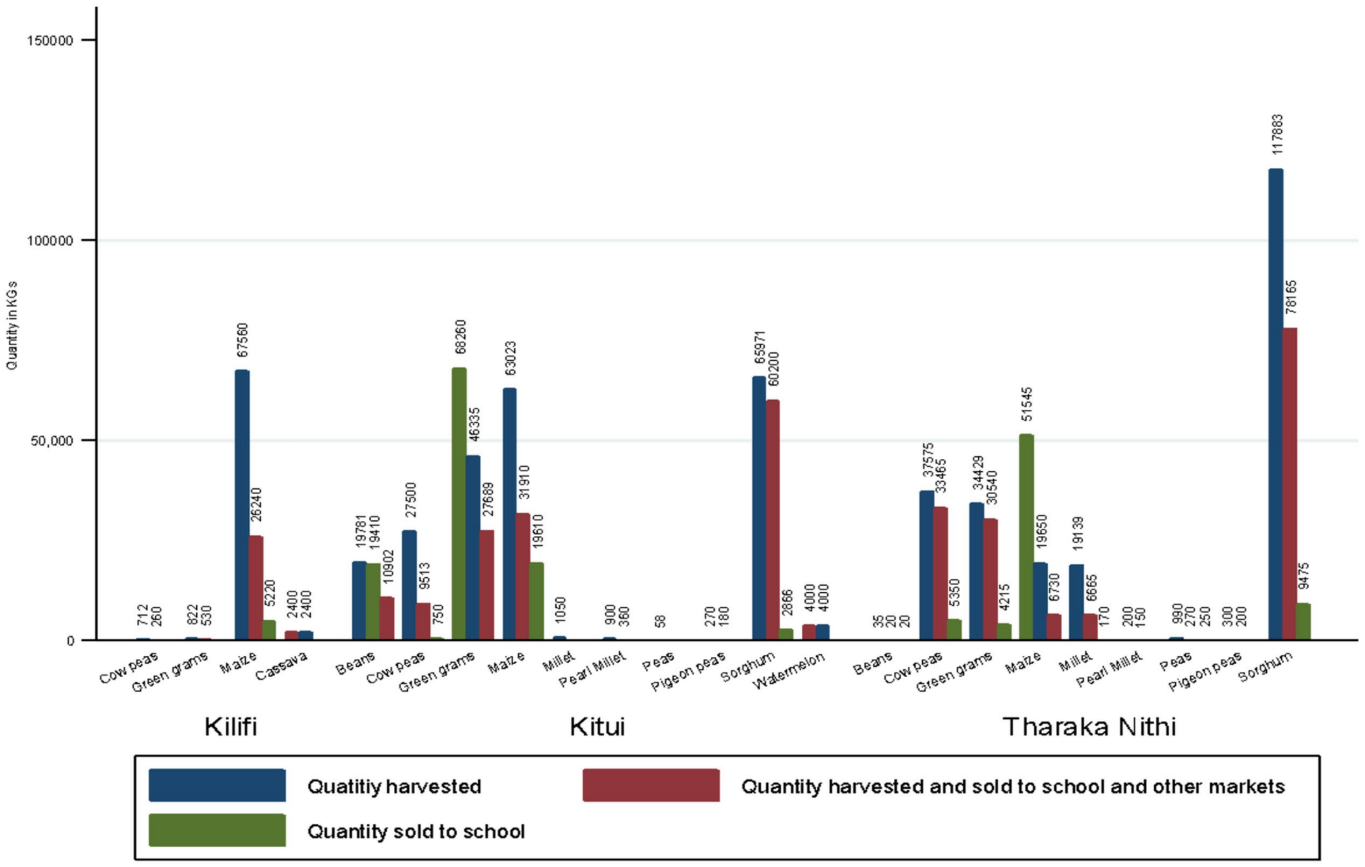
Quantity of crops produced and sold last season by counties.

During the season, SHFs cultivated an average of 3.5 acres of land each (SD: 3.2, Range: 0–27.0) on which they produced a variety of crops. The majority (60.5%) produced green grams, maize (59.5%), cowpeas (47.4%), sorghum 118 (31.2%) and beans 83 (21.9%). Sorghum recorded the highest production (183,854 Kg) followed by maize (150,233 kg), green grams (81,856 kg) and cowpeas (65,787 kg). The crops that were mostly sold to other markets were sorghum (138,365 Kg), maize (64,880 kg) and green grams (58,759 kg) (Figure 1). However, the crops that were sold to schools either individually or through their FBOs were maize (76,375 Kg), green grams (72,475 Kg) and beans (19,430 kg) (Figure 1). Notably, the quantities of maize, green grams and beans supplied to schools exceeded the quantity produced by participants. This could imply that the SHFs may have aggregated some produce from other farmers to achieve the quantities required for schools. The crop with the highest total sales was sorghum (KES 5,965,950) followed by green grams (KES 4,416,910). Production and marketing of food crops to schools and other markets within the study counties by SHFs is presented in Figure 2.

### Smallholder farmer access to HGSFP market

At least 22.8% of SHFs accessed schools under the HGSFP and sold some of their produce (Table 2). More SHFs from Tharaka Nithi County (37.4%) accessed the HGSFP market compared to Kitui County (17.1%) and Kilifi County (16.4%). The main channel that was used to sell produce to schools was FBOs (61.6%) led by farmers in Tharaka Nithi County (85%). Most of the farmers who did not sell to schools reported a lack of surplus as their main reason (53.2%), and failure to win school tenders (15.9%). Nonetheless, 64.8% of SHFs sold their produce to other markets. SHFs from Tharaka Nithi County had the greatest access to other markets (96.3%) followed by Kilifi County (60%) and Kitui County (50.5%). The main challenge experienced by farmers when selling their produce was low prices in the available markets (50.9%) and high cost of transport/poor infrastructure (23.6%). The latter was the biggest challenge among farmers in Kilifi County (46.2%) (Table 2). The prices of produce were negotiated based on the market rates. However, half of the SHFs (50.5%) reported that the prices offered were not fair as compared to the market rates (Table 2).

**Table 2:** Crop marketing and marketing channels used by smallholder farmers.

Factors	Overall no. of farmers	No. of farmers by county		
		Kitui	Tharaka Nithi	Kilifi
Sell to school under HGSFP (%)				
Yes	86 (22.8)	37 (17.1)	40 (37.4)	9 (16.4)
No	292 (77.3)	179 (82.9)	67 (62.2)	46 (83.6)
Channels used to sell to school, *n* = 77 (%)				
Broker	8 (9.3)	6 (16.2)	2 (5.0)	0
Farm gate	5 (5.8)	4 (10.8)	1 (2.5)	0
Farmer organization	53 (61.6)	16 (43.2)	34 (85.0)	3 (5.5)
Mobile phone	16 (18.6)	7 (18.9)	3 (7.5)	6 (10.9)
Open market	1 (1.2)	1 (2.7)	0	0
Other	3 (3.5)	3 (8.1)	0	0
Reason farmer did not sell to school (%)				
Home consumption	25 (6.6)	10 (4.6)	1 (0.9)	14 (25.5)
No surplus	201 (53.2)	144 (66.7)	33 (30.8)	24 (43.6)
Didn’t win tender	60 (15.9)	20 (9.3)	27 (25.2)	13 (23.6)
Poor quality	1 (0.3)	1 (0.5)	0	0
Price not attractive	10 (2.7)	7 (3.2)	3 (2.8)	0
Other	81 (21.4)	34 (15.7)	43 (40.2)	4 (7.3)
Sell to other markets (%)				
Yes	245 (64.8)	109 (50.5)	103 (96.3)	33 (60.0)
No	133 (35.2)	107 (49.5)	4 (3.7)	22 (40.0)
Ways used to decide price (%)				
Price decided by FBO	43 (11.4)	19 (8.8)	21 (19.6)	3 (5.5)
Price offered by buyer	104 (27.5)	57 (26.4)	44 (41.1)	3 (5.5)
Negotiated price based on market rates	136 (36.0)	68 (31.5)	39 (36.5)	29 (52.7)
Other	90 (23.8)	69 (31.9)	1 (0.9)	20 (36.4)
Was price fair (%)				
Yes	187 (49.5)	71 (32.9)	87 (81.3)	29 (52.7)
No	191 (50.5)	145 (67.1)	20 (18.7)	26 (47.3)
Experience difficulties when selling (%)				
Yes	165 (43.7)	96 (44.4)	56 (52.3)	13 (23.6)
No	213 (56.4)	120 (55.6)	51 (47.7)	42 (76.4)
Difficulties experienced when selling, *n* = 152 (%)				
Accessing the market	26 (15.8)	14 (14.6)	11 (19.6)	1 (7.7)
High cost of transport/poor infrastructure	39 (23.6)	23 (24.0)	10 (17.9)	6 (46.2)
Lack of support from FBO	2 (1.2)	2 (2.1)	0	0
Low prices in available markets	84 (50.9)	52 (54.2)	31 (55.4)	1 (7.7)
Lack of price information	4 (2.4)	1 (1.0)	2 (3.6)	1 (7.7)
Inability to meet quality requirements	3 (1.8)	1 (1.0)	0	2 (15.4)

### Marketing by farmer based organizations and experience with digital marketing platforms

A total of 92 FBOs participated in the study. This comprised of 39 (54.2%) FBOs in Kitui County, 33 (45.8%) in Tharaka Nithi County and 20 (21.7%) in Kilifi County. More than half of the FBOs (53.3%) reported that they usually sell produce on behalf of members while 22.8% connect their members to other markets and 15 (16.3%) do not market their members’ produce. The main markets accessed by FBOs were traders/brokers (44.9%), open-air markets (18.4%), schools in the HGSF programme (18.4%), food processors 5 (10.2%), and other primary and secondary schools 4 (8.2%) (Table 3). The most marketed crops by FBOs were sorghum (32.6%) green grams (31.5%) and maize (28.3%). Sorghum recorded the highest quantity sold by FBOs (1,089,450 Kg) followed by millet (356,400 kg), green grams (75,750 kg) and cowpeas (75,250 kg) mostly by FBOs in Tharaka Nithi County. Maize registered the highest sales in Kitui (87,620 kg) and Kilifi Counties (64,410 kg) which were mainly for HGSFP schools in the respective counties.

**Table 3:** Marketing by farmer-based organizations and experience with digital marketing platforms.

Factor	Overall	By county		
		Kitui	Tharaka Nithi	Kilifi
FBO market members produce (%)				
Sell on behalf of members	49 (53.3)	19 (48.7)	18 (54.6)	12 (60.0)
Connect members to other markets	21 (22.8)	7 (18.0)	10 (30.3)	4 (20.0)
Does not market members’ produce	15 (16.3)	6 (15.4)	5 (15.2)	4 (20.0)
Others	7 (7.6)	7 (18.0)	0	0
Markets FBO sell members’ produce, *n* = 49 (%)				
Open air markets	9 (18.4)	4 (21.1)	1 (5.6)	4 (33.3)
HGSF schools	9 (18.4)	3 (15.8)	2 (11.1)	4 (33.3)
Other schools (primary and secondary)	4 (8.2)	2 (10.5)	2 (11.1)	0
Brokers	22 (44.9)	9 (47.4)	9 (50.0)	4 (33.3)
Food processors, e.g., millers	5 (10.2)	1 (5.3)	4 (22.2)	0
Used MPP to bid to supply to school (%)				
Yes	56 (60.9)	14 (35.9)	26 (78.8)	16 (80.0)
No	36 (39.1)	25 (64.1)	7 (21.1)	4 (20.0)
Did FBO qualify for the tender, *n* = 56 (%)				
Yes	21 (37.5)	5 (35.7)	9 (34.6)	7 (43.8)
No	35 (62.5)	9 (64.3)	17 (65.4)	9 (56.3)
FBO sold food to schools without using MPP (%)				
Yes	30 (32.6)	11 (28.2)	13 (39.4)	6 (30.0)
No	62 (67.4)	28 (71.8)	20 (60.6)	14 (70.0)
Advantages of mobile tendering over traditional tendering, (%)				
It is faster	43 (76.8)	11 (78.6)	19 (73.1)	13 (65.0)
Cheaper to use	23 (41.1)	7 (50.0)	9 (34.6)	7 (35.0)
Offers access to more markets	32 (57.1)	11 (78.6)	14 (53.8)	7 (35.0)
More transparent in tendering	25 (44.6)	5 (35.7)	16 (61.5)	4 (20.0)
Access to market information	25 (44.6)	7 (50.0)	9 (34.6)	9 (45.0)
Other	1 (1.8)	0	0	1 (5.0)
Challenges experienced when using MPP, (%)				
Limited knowledge on its usage	16 (28.6)	6 (42.9)	7 (26.9)	3 (15.0)
Poor network coverage	18 (32.1)	7 (50.0)	9 (34.6)	2 (10.0)
Expensive to use	10 (17.9)	7 (50.0)	1 (3.8)	2 (10.0)
Insufficient training	31 (55.4)	6 (42.9)	21 (80.8)	4 (20.0)
Others	20 (35.7)	2 (14.3)	8 (30.8)	10 (50.0)

Regarding the use of the MPP, 60.9% of the FBOs used MPP to bid for school tenders out of which 37.5% won the tenders. The most participating FBOs were in Kilifi County (80%) followed by Tharaka Nithi County (78.8%). More FBOs in Kilifi County (43.8%) managed to sell food to HGSFP schools using the MPP. However, 32.6% of the FBOs sold food to the schools without using the MPP majority of which were from Tharaka Nithi County (39.4%). The use of the MPP for procurement of school meal items was rated as faster to use (76.8%), offered access to more markets (57.1%) and was more transparent (44.6%). The main challenge experienced while using the MPP was insufficient training 55.4%) (Table 3).

### HGSFP schools and school meals procurement

#### Type of food and quantity procured by HGSFP schools

**Figure 3:**
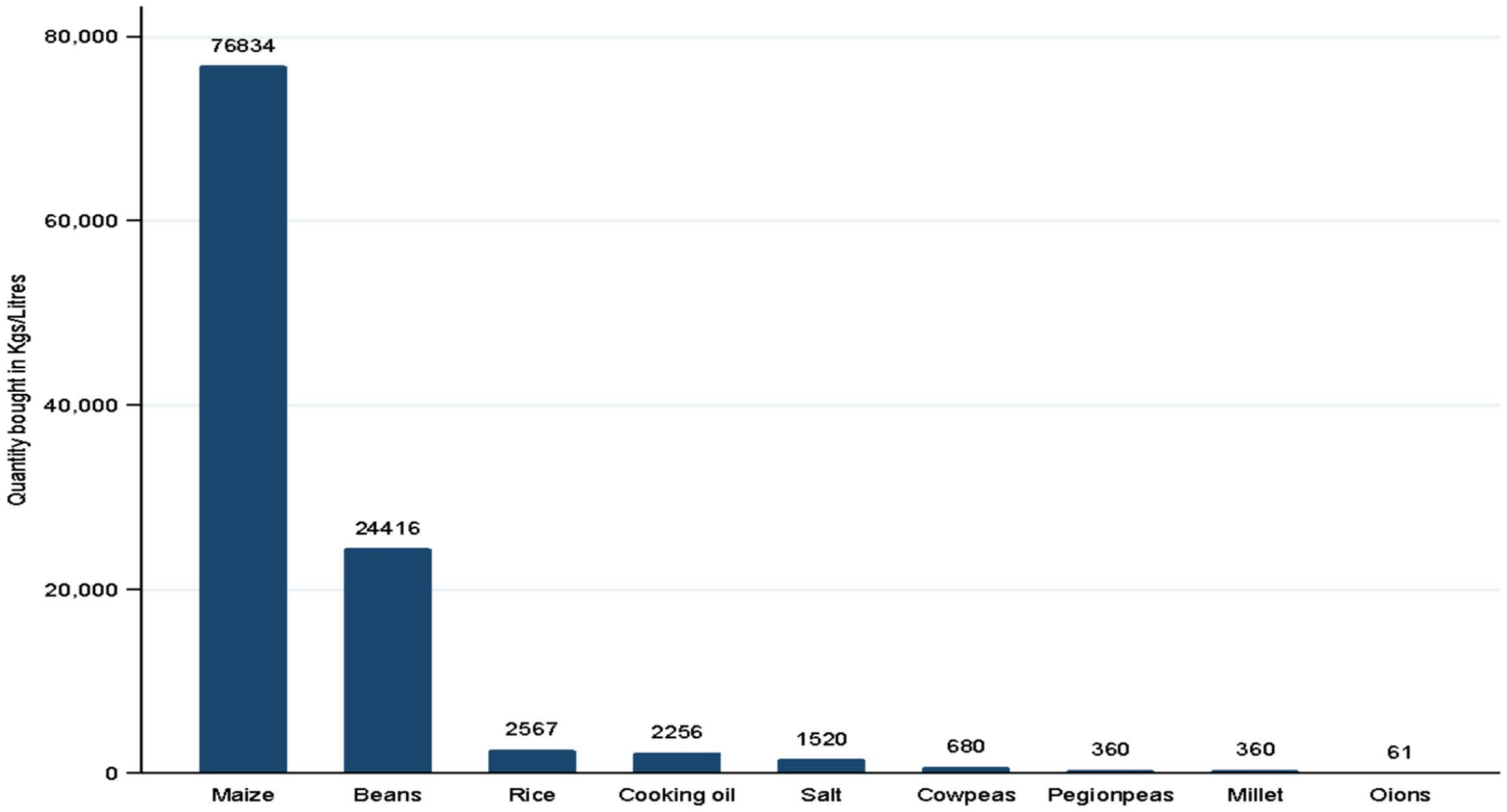
Quantities of food bought by schools in the last procurement cycle.

From the study, maize and beans were the main food items bought for school meals at (92.9%) and (91.4%) respectively, followed by salt (71.4%) and cooking oil (70%). The total quantities of food items procured by schools are shown in Figure 3 where maize (76,834 kg) and beans (24,416 kg,) recorded the highest amounts.

#### Procurement process in schools

The majority of schools (91.4%) published tenders for food commodities during the year out of which 65.6% did manual tendering while 26.5% tendered through the MPP. More schools in Kitui County did manual tendering (79.2%) while tendering through the MPP was more popular in schools in Tharaka Nithi County (36.4%) (Table 4). Further, the teachers reported that it took more than 10 days for most of the schools (67.2%) to receive food from suppliers after publishing the tenders (Table 4).

**Table 4:** Procurement in the HGSFP schools.

Factors	Overall	By county		
		Kitui	Tharaka Nithi	Kilifi
Whether the school placed a tender in the last procurement cycle				
Yes	64 (91.4)	24 (88.9)	22 (91.7)	18 (94.7)
No	6 (8.6)	3 (11.1)	2 (8.3)	1 (5.3)
Type of tender (*n* = 64)				
Manual	42 (65.6)	19 (79.2)	14 (63.6)	9 (0.5)
Through MPP	17 (26.5)	5 (20.8)	8 (36.4)	4 (22.2)
Other	5 (7.8)	0	0	5 (27.8)
Days from publishing tender to supply (*n* = 64)				
Less than 5 days	8 (12.5)	3 (12.5)	5 (22.7)	0
5–10 days	13 (20.3)	5 (20.8)	5 (22.7)	3 (16.7)
More than 10 days	43 (67.2)	16 (66.7)	12 (54.6)	15 (83.3)
Days from supply to payment of the supplier (*n* = 64)				
Less than 5 days	43 (67.2)	17 (70.8)	18 (81.8)	8 (44.4)
5–10 days	15 (23.4)	6 (25.0)	3 (13.6)	6 (33.3)
More than 10 days	6 (9.4)	1 (4.2)	1 (4.6)	4 (22.2)
Communication to the winning bidder (*n* = 64)				
Through SMS	10 (15.6)	8 (33.3)	0	2 (11.1)
Through a telephone call	45 (70.3)	12 (50.0)	18 (81.8)	15 (83.3)
Through a visit to the supplier	2 (3.1)	2 (8.3)	0	0
Through the MPP (automatically)	2 (3.1)	2 (8.3)	0	0
Other	5 (7.1)	0	4 (22.2)	1 (5.6)
Whether school communicated with the unsuccessful bidders (*n* = 64)				
Yes	33 (51.6)	13 (54.2)	14 (63.6)	6 (33.3)
No	31 (48.4)	11 (45.8)	8 (36.4)	12 (66.7)
School tendered through MPP in 2023 procurement cycle (*n* = 64)				
Yes	53 (82.8)	19 (79.2)	19 (86.4)	15 (83.3)
No	11 (17.2)	5 (20.8)	3 (13.6)	3 (16.7)
Whether training offered on MPP was adequate (*n* = 64)				
Yes	35 (54.7)	8 (33.3)	15 (68.2)	12 (66.7)
No	29 (45.3)	16 (66.7)	7 (31.8)	6 (33.3)

The mode of communication to the winning bidders for most schools was through telephone calls, (70.3%) and short message service (SMS) (15.6%). More than half of the schools also communicated with the unsuccessful bidders (51.6%). Overall, 53 schools (82.8%) reported that they had ever published tenders on the MPP led by schools in Tharaka Nithi County (86.4%) (Table 4).

## Discussion

The study was conducted in three counties; Kitui, Tharaka Nithi and Kilifi among SHFs, FBOs, and HGSFP schools. The purpose of the study was to explore the extent to which SHFs were able to access and participate in HGSFP, the type of food crops supplied to participating schools and the use of the MPP in the HGSFP procurement process.

### Smallholder farmer access to HGSFP market

SHFs across the three counties produced different kinds of crops including sorghum, green grams, maize and beans with the highest production recorded for sorghum. The quantities of maize and beans sold to schools exceeded those produced by SHFs. This implies that SHFs aggregated some produce from other farmers to achieve the quantities that were required for schools. Aggregation of produce by SHFs enables them to raise large volumes of produce to supply to formal markets such as schools and is also a platform for access to inputs such as seeds and fertilizers.

A small proportion of SHFs (22.8%) sold their produce to schools. Individual SHF access to schools could be limited because SHFs are encouraged to join FBOs to qualify as school food suppliers. FBOs were the main channel when selling to schools and other markets. The HGSFP implementation guidelines require SHFs to belong to FBOs to help them to aggregate their produce and fulfill the quantities needed by the HGSFP school market (28). In addition, SHFs are encouraged to join FBOs so that they can benefit from collective marketing activities, farm inputs and capacity-building activities (6). Further, a study by Othman showed that participation of SHFs in collective marketing led to increased productivity and improved incomes (5) and could lead to better quality of food (7). The limited SHF access to HGSFP market concurs with a study done in Kwale County by Karisa and Orodho (20) which reported that SHFs were not benefitting from the HGSFP market in the county. However, this study shows a gradual penetration of SHFs through FBOs into the school meals market which concurs with a study by Espejo that observed that buying food from SHFs for the HGSFP market would be gradual and highlighted the need to create an enabling environment for SHFs to access the school market (17).

### Farmer-based organizations’ access to the HGSFP market

The study revealed that half of the participating FBOs (53.3%) sold produce on behalf of their members mostly to traders, open-air markets and schools. The majority participated in the tendering procedures for school meals by publishing bids for school meals tenders where 37.5% won and sold food to the schools through the MPP. Notably, 32.6% sold food to schools manually. While the program emphasizes local sourcing of food through FBOs, it is evident that they were underutilized or lacked the capacity to supply the school market. Consequently, local traders supplied food to most of the schools. The study established that the supply of food to schools by FBOs was low because of the drought that was experienced in Kenya from 2020 to 2022 leading to a loss of harvest during the period. As such, the SHFs used up the little harvest for home consumption as there was little or no surplus to aggregate and sell to schools or other markets. These findings concur with a study by Okumu and Muhingi in Machakos County (19) where food production by SHFs was too low to support school feeding and home consumption hence, traders would source cereals for school meals from outside the county. Similarly, a study of HGSFP schools in Kwale County (20) reported higher food purchases from traders compared to SHFs. In this study, unfavorable prices offered by schools and the high transport cost also discouraged some FBOs and their members from selling their produce to schools.

### HGSFP schools’ food procurement

The average amount spent on food per school was KES 148,878.10, with a wide range (min: KES 0, max: KES 503,000). The head teachers from participating schools reported that cash disbursements to schools for the meals were mostly insufficient, were done late into the year and some schools missed out altogether. This finding agrees with a study of HGSFP schools in Kwale County (20) and is contrary to the guidelines for HGSFP design and implementation that calls for timely release of funds for the programme.

The most procured food items for school meals across the three counties were maize and beans which were bought by 92.9 and 91.4% of schools, respectively. The schools procured a total of 76,834 kg of maize and 24,416 kg of beans across all counties in the 2022 procurement cycle. Cooking oil and salt are mandatory items in the food basket and were included in the purchases for school meal items. The Kenya HGSFP Implementation Guidelines (28) provide a minimum food basket comprising cereal (150 grams), pulses (40 grams), vegetable oil (5 mL) and iodized salt (5 grams) per child. The guideline further states that school meal ratios should contain sufficient amounts of carbohydrates, proteins and fats and should include sources of micronutrients such as fruits and vegetables. The overwhelming focus on maize and beans for school meals in the study is a pointer to the lack of dietary diversity and highlights the need to ensure that pupils receive a wider range of essential nutrients. The finding is also contrary to recommendations by Pastorino et al. (29) that promote the provision of diverse foods that are local, seasonal and nutrient-rich to address nutritional deficiencies among school-going children in areas with micronutrient deficiencies. This is because the consumption of fruits, vegetables, legumes and fatty fish in school diets is limited in many countries (29). This means that schools should try and provide nutritious diets that are practical and feasible based on cash allocation as well as nutritionally appropriate. This finding varies slightly with a study conducted by Kimwele et al. in Makueni County which reported that children in HGSFP schools had better dietary diversity compared to children in non-HGSFP schools (22).

In the study, there was a mismatch between the crops that were produced by the SHFs and those that were procured by schools. It was noted that the production of beans was low despite the overreliance on the pulse as a source of protein in school meals. In addition, sorghum was produced in large quantities, especially in Tharaka Nithi and Kitui Counties yet none of the schools reported using sorghum in their meals. This is contrary to the Agriculture-related objectives of HGSFP which include linking school feeding to local agricultural production (28). This means that schools should be able to utilize other locally available foods that are produced in high quantities in the project area such as sorghum, green grams, and cowpeas by developing recipes that incorporate these food crops. This may encourage diversity in school meals and lead to savings occasioned by purchasing foods that are scarce in the locality. Further, the inclusion of drought-resistant crops and foods that are adaptive to local conditions in school menus will help achieve planet-friendly school meals (29).

Concerning the procurement process, most schools (91.4%) published tenders for the procurement of food items although manual tendering (65.6%) was higher compared to the use of MPP (26.5%). Studies show that existing mobile technology innovations in school meals mainly deal with tracking pupils’ attendance and food distribution but less with linking SHFs to schools through food procurement. The MPP is therefore a novel innovation that can provide real-time data on the procurement processes of HGSFP from publication of food tenders, selection of winning bidders up to delivery of food and payment. Further, digitalization of the HGSFP procurement process could lead to enhanced accountability, transparency and inclusivity of FBOs and traders. In addition, digital innovations have been shown to support SHFs in dealing with factors that exclude them from formal markets (30) while supporting them in decision-making, increased production and incomes, and more access to information and services including access to markets (31).

In the study, traders/brokers were the dominant suppliers of food items for school meals followed by FBOs. This finding is consistent with research on Ghana’s HGSFP (32) which indicated that traders were the largest procurement channel among the caterer-designed HGSFP. Traders can supply because they source their products from within and outside the local communities. In the case of food deficits due to droughts, traders would traverse other counties with higher production capacity and procure food commodities in bulk thus saving on costs.

## Conclusion

SHFs and FBOs gained access and participated in the HGSFP albeit to some extent. However, local procurement opportunities through FBOs were underutilized as HGSFP schools purchased most of their food requirements from traders/brokers. FBOs and SHFs may require further support and agricultural extension to build their capacity to a level where they can competently participate in school meal tenders and supply the necessary quantities of food commodities. This support should help the SHFs to increase, adapt, and diversify production based on environmentally friendly production services to meet HGSFP requirements. Training on the procurement systems of HGSFP will further build SHFs’ capacity to access the market.

Food procurement through the MPP was embraced by schools and FBOs and should be inclusive of all market players including traders as they are significant players in the school meals supply chain. Adequate training is required for all the stakeholders involved in the programme to make the digital platform function effectively. Diversification of school diets should be encouraged for schools to benefit from local production.

## Data Availability

The raw data supporting the conclusions of this article will be made available by the authors, without undue reservation.

## References

[1] FAO, “Kenya at a glance,” FAO Kenya (2024). Available: https://www.fao.org/kenya/fao-in-kenya/kenya-at-a-glance/en/

[2] Statista, “Agricultural land in Kenya from 2010 to 2019”. (2024). Available at: https://www.statista.com/statistics/1287332/agricultural-land-in-kenya/

[3] Republic of Kenya, “Agricultural Sector Development Strategy 2010-2020,” Ministry of Agriculture. (2010).

[4] Kamara A Conteh A Rhodes ER Cooke RA. The relevance of smallholder farming to african agricultural growth and development. African J Food, Agric Nutr Dev. (2019) 19:14043–65. doi: 10.18697/AJFAND.84.BLFB1010

[5] Othman S Oughton E Garrod G. Significance of farming groups for resource access and livelihoods improvement of rural smallholder women farmers. Dev Pract. (2020) 30:586–98. doi: 10.1080/09614524.2020.1764502

[6] Kumar V Wankhede KG Gena HC. Livelihood of farmers on sustainable basis. Am J Educ Res. (2015) 3:1258–66. doi: 10.12691/education-3-10-8

[7] African Union Commission and African Union Development Agency - NEPAD., “AUDA-NEPAD Guidelines for the Design and Implementation of Home Grown School Feeding Programmes in Africa.,” AUDA-NEPAD. Midrand, South Africa., (2022), Accessed on Nov. 11, 2024. Available at:https://www.nepad.org/sites/default/files/resourcefiles/Guidelines for HGSF Implementation_EN.pdf

[8] FAO and WFP, “Home-grown school feeding. Resource framework. (2018). Accessed on Jun. 20, 2024. Available at:https://www.wfp.org/publications/home-grown-school-feeding-resource-framework

[9] Prifti E Grinspun A. Impact evaluation of the home grown school feeding and conservation agriculture scale-up programmes in Zambia. Rome: FAO (2021).

[10] Margrethe B. A. Haug R., “Home Grown School Feeding in Low-In come Countries: Harvesting Benefits for Smallholder Farmers,” (2022). Available at: https://www.nmbu.no/en/faculty/landsam/department/noragric

[11] Partnership for Child Development, “Sustainable School Feeding Programmes: A Guidance Note to Develop a National Sustainability Strategy,” WFP, The World Bank and PCD London. (2012).

[12] WFP, “State of School Feeding Worldwide 2022,” WFP. (2022).

[13] World Food Programme, “School Meals Programme in Kenya” WFP. (2018).

[14] FAO, “Food systems solutions to help end hidden hunger governance and accountability for food fortification.” (2024). Available at: https://cdn.who.int/media/docs/default-source/anaemia/areacop-webinar

[15] Partnership for Child Development. Home grown school feeding. London: PCD (2012).

[16] Bhalla G., “The role of social protection in strengthening local food systems and inclusive rural transformation,” FAO. (2023).

[17] Espejo F Burbano C Galliano E. Home - grown school feeding: A framework to link school feeding with local agricultural production. Rome Italy: WFP. (2009).

[18] Drake L Woolnough A Burbano C Bundy D Global School Feeding Sourcebook. Lessons from 14 countries. London: Imperial College Press (2016).

[19] Bonareri G. Muhingi Wilkins N., “Homegrown school feeding Programme (HGSFP) and agricultural production by small-scale farmers in Mwala Sub-County, Machakos County, Kenya” (2021). Available at: www.jriiejournal.com

[20] Karisa K. S. Orodho J. A., “Assessment of home grown school feeding Programme (HGSFP) theory in Kinango Sub-County, Kwale County, Kenya,”(2014). Available at: www.iosrjournals.org

[21] Kiilu RM. Status of school feeding programme policy initiatives in primary schools in Machakos County, Kenya. African Educ Res J. (2019) 7:33–9. doi: 10.30918/AERJ.71.18.107

[22] Kimwele A.M Ochola S. A Mugambi M. N, “Influence of Homegrown school feeding Programme on dietary diversity among school children 6–13 years of age in Makueni county, Kenya,” (2021). Euro J Health Sci, doi: 10.47672/ejhs.678, 6, 57–72.

[23] KNBS, “VOLUME IV KPHC 2019,” Kenya Population and Housing Census 2019. the Publisher is Kenya National Bureau of Statistics (KNBS). (2019).

[24] MOALFC, “Climate risk profile for Kitui County. Kenya County climate risk profile series. The Ministry of Agriculture, livestock, fisheries and cooperatives, Nairobi. Kenya,” Nairobi, Kenya: Ministry of Agriculture, Livestock, Fisheries and Cooperatives. (2021).

[25] County Government of Kilifi, “County Integrated Development Plan 2018 - 2022,” Council of Governors (COG). (2018).

[26] County Government of Tharaka Nithi, “Third county integrated development plan 2023 - 2027,” (2023). Available at: www.tharakanithi.go.ke

[27] Yamane Y., “Mathematical formulae for sample determination,” 2nd edition. Harper and Row. New York. (1967).

[28] Republic of Kenya, “Home grown school meals Programme implementation guidelines,” Nairobi, Kenya: Ministry of Education. (2016).

[29] Pastorino S. Darren H. Linda S. Samantha O. Morris Kate, School meals and food systems: rethinking the consequences for climate, environment, biodiversity and food sovereignty. Reseach Consortiun for School and Nutrition. January 2024 (2023).

[30] Schulz C., “The inclusiveness of digital innovations in the south African agriculture system closing the inequality gap through innovation,” (2019).

[31] Malabo Montpellier Panel, Byte by Byte: Policy Innovation for Transforming Africa’s Food System with Digital Technologies Malabo Montpellier Panel. (2019). Available at: https://hdl.handle.net/10568/146526

[32] Gelli A.., School meals as a market for smallholder agriculture. Experimental evidence from Ghana. (2024). Available at: https://papers.ssrn.com/sol3/papers.cfm?abstract_id=3936650

